# Silybin Prevents Prostate Cancer by Inhibited the ALDH1A1 Expression in the Retinol Metabolism Pathway

**DOI:** 10.3389/fcell.2020.574394

**Published:** 2020-08-31

**Authors:** Ying Jiang, Hanbing Song, Ling Jiang, Yu Qiao, Dan Yang, Donghua Wang, Ji Li

**Affiliations:** ^1^College of Basic Medicine, Heilongjiang University of Chinese Medicine, Harbin, China; ^2^The First Affiliated Hospital, Heilongjiang University of Chinese Medicine, Harbin, China; ^3^Department of General Surgery, General Hospital of Heilongjiang Province Land Reclamation Bureau, Harbin, China

**Keywords:** prostate cancer, ALDH1A1, silybin, metabolism pathway, biomarker

## Abstract

**Background:**

Silybin was known to exert inhibition in prostate cancer, but the underlying mechanism remained largely unknown. This study was designed to find out the potential target of Silybin on prostate cancer and explore the relative mechanisms.

**Methods:**

Firstly, we screened the possible targets of Silybin through the PubChem database and Subpathway – GM. Then DU145 cells were transferred to investigate the correction about related targets, magnetic bead sorting and flow cytometry were used to sort and identify the cells. Proliferation, migration and invasion ability of DU145 cells were detected by MTT assay, Transwell assay, plate clonality and sphere formation assay. BALB/c nude mice were constructed models with implanted sarcoma and measured the tumor volume every 5 days as wells tumor weight. The levels of proteins were detected by Western blot and immunocytochemistry. RT-PCR was selected to test the expression of protein’s mRNA.

**Results:**

It was screened out the ALDH1A1 was highly correlated with subpathways of the Silybin risk metabolic pathway. And ALDH1A1 expression was positively correlated RARα with Ets1 by interfering with the ALDH1A1 gene. Importantly, ALDH1A1(+) cells showed proliferation, migration and invasion ability. In addition, it showed that Silybin exerted the inhibition on prostate cells by suppressed the proliferation, migration and invasion ability of cells *in vitro* experiment. Silybin also reduced the tumor volume and weight. And Silybin displayed obviously reduced the proteins and mRNA of ALDH1A1, RARα, Ets1 and MMP9 expressions.

**Conclusion:**

Our results indicated that Silybin showed inhibition of prostate cancer and the mechanism was involving with downregulating ALDH1A1 expression, thereby inhibiting the activation of RARα and preventing the activation of Ets1 to inhibit the growth and invasion of prostate cancer.

## Introduction

Prostate cancer is one of the malignant tumors that endanger the health of men around the world and the incidence is getting more common in recent years (Arcangeli et al., 2012; Kimura and Egawa, 2018; Lin et al., 2019; Qu et al., 2019; Zeng et al., 2019; Zhou et al., 2019). At present, the common treatment options were radical prostatectomy, chemotherapeutic drugs, anti-androgenic therapies, immunotherapy and so on (Ahmad et al., 2017; Gamat and McNeel, 2017; Lawrence et al., 2018; Liao et al., 2018; Liu et al., 2018; Tang et al., 2018; Ji et al., 2019; Han et al., 2020). However, risk factors of these treatments, such as permanent urinary incontinence, peripheral neuropathy and high cost, limited the applications (Green et al., 2015). Therefore, it was of great theoretical and practical significance to find effective treatment for prostate cancer.

Silybin, a mixture of flavonolignan and flavonoid polyphenolic compounds extractable from milk thistle seeds, had high antioxidant and anticancer properties, which caused broad-spectrum efficacy against cancer (Jahanafrooz et al., 2018; Di Costanzo and Angelico, 2019). Actually, Silybin had been widely investigated for anti-cancer efficacy in a broad range of cancers models such as cervical cancer, liver cancer and prostate cancer especially (Varghese et al., 2005; Ting et al., 2013). Some research findings revealed that Silybin could inhibit the secretion of angiogenic factors and targeting the responses of endothelial angiogenic factors (motility, proliferation, survival, and differentiation) effectively for the treatment of prostate cancer cells (Singh et al., 2008). Evidence suggested that ALDH1A1 was significantly associated with prostate cancer risk and which could be a prostate cancer stem cells-related marker (Li et al., 2010). Furthermore, Silybin could downregulated the expression of ALDH1A1 to alleviate the process of neck squamous cell carcinomas (Hellsten et al., 2011). And it had also been indicated that Silybin could significantly induced the expression of matrix metalloproteinases (MMPs) to prevent the tumor metastasis *in vitro* and *vivo* studies (Deep and Agarwal, 2010). Although Silybin could inhibit prostate cancer cell proliferation, its effect on stem cell-like cells from prostate cancer is unclear. Presuming finding the potential targets on prostate stem cancer would have great prospect.

Recently, it had become one of the most important focuses to screen drug targets by bioinformatics method which was beneficial to improve the safety and reliability of drug application (Gao et al., 2018; Lai et al., 2018; Cheng et al., 2019; Feng, 2019; Su et al., 2019). For example, Huang et al. (2017) identified some hub genes of prostate cancer through an integrated bioinformatics approach and Foj and Filella (2019) identified of potential miRNAs biomarkers for high-grade prostate cancer by integrated bioinformatics analysis. As a matter of fact, the development of bioinformatics technology had not only made great achievements in disease mechanism research and diagnosis and treatment (Han et al., 2018; Zhang et al., 2020), but also provided new concepts and means in the way of finding new drug action targets and the preparation process, which greatly promoted the pace of medical research and development from both theoretical and technical aspects (Du et al., 2018; Di et al., 2019; Sun et al., 2019; Wu et al., 2019; Cheng et al., 2020).

In this study, we firstly obtained potential targets of Silybin for prostate cancer by bioinformatics method and then confirmed the related pathology on proliferation, migration and invasion of prostate stem cancer. And we further explored the mechanism of Silybin in inhibiting prostate cancer by the mechanism *in vitro* and *vivo*.

## Materials and Methods

### Target-Based Screening by Bioinformatics Analysis

We found the possible targets of Silybin through the PubChem database^1^, and obtained 26 active targets and then screen for the highly correlated target by inputting the NCBI-Gene ID of each gene into Subpathway – GM^2^ (Li et al., 2013).

### Chemicals and Regents

Silybin (HY-13748) was bought from Medchemexpress (NJ, United States). RPMI-1640 (31800-014) was purchased from Gibco (Shanghai, China). Anti-Biotin MicroBeads (130-105-637) was obtained from Miltenyi Biotechnology Co. Ltd. (Kölner, German). Biotin-labeled ALDH1A1 antibody (NBP2-54422B) was got from Novus Biologicals (CO, United States). Polyclonal antibodies of ALDH1A1 (WL02762), Ets1 (WL02523), MMP-9 (WL03096) were bought from Wanlei Biotechnology Co. Ltd. (Wuhan, China) and RARα (10331-1-AP) were bought from proteintech group, Inc. (Wuhan, China). RNase inhibitor (RP5602), 2 × Power Taq PCR MasterMix (PR1702) and Super m-mlv reverse transcriptase (PR6502) were got from BioTeck Biotechnology Co. Ltd (Beijing, China). Retinoic Acid (ATRA, R106320) was bought from Aladdin (Shanghai, China).

### *In vitro* Experiment

#### Cell Treatment and Transfection

DU145 was obtained from bought from Wanlei Biotechnology Co. Ltd. (Wuhan, China). DU-145 cells were cultured with RIMI-1640 containing 10% FBS at 37°C and 5% CO_2_ in the incubator. Using trypsin to digest DU-145 cells in logarithmic growth stage and disperse them into a cell suspension, and dilute the cell density to 2 × 10^5^/mL. The cells were seeded in 96-well plates at the density of 2 × 10^4^/well. Lipofectamine^TM^ 2000 was transfected randomly into DU-145 cells. The experience consisted of five groups: Untranslated group, negative matched control transfection group and transfection group (siRNA-1, siRNA-2, siRNA-3) (Table 1). According to the ALDH1A1 gene sequence to determine the start site of gene transcription, and the specific interference sites were listed in the Supplementary Figure S1. Successful transfections were determined using quantitative RT-PCR or the western blot assay. ALDH1A1+ cells were treated with DMSO, Silybin (50, 100 150, 200 μM) and siRNA-ALDH1A1 for 48 h.

**TABLE 1:** siRNA sequence of ALDH1A1.

Name	Sequence
ALDH1A1-homo-591	GGCUGAUUUAAUCGAAAGATTUCUUUCGAUUAAAUCAGCCTT
ALDH1A1-homo-924 ALDH1A1-homo-1288	CCACGUGGCAUCUUUAAUATTUAUUAAAGAUGCCACGUGGTT GAGCGGGCUAAGAAGUAUATTUAUACUUCUUAGCCCGCUCTT
Negative Control	UUCUCCGAACGUGUCACGUTTACGUGACACGUUCGGAGAATT

#### Magnetic Bead Cell Sorting

Cells with a cell density of 1 × 10^8^/mL were placed in a test tube and incubated in biotin-labeled ALDH1A1 antibody for 5 min. Centrifuged for 10 min at 300 *g* and washed twice with PBS. After obtaining the reclosed cells, the MS separation column was placed in the magnetic field of the MACS separator and rinsed with 500 μL Buffer. The 500 μL Buffer cell suspension was passed through the separation column and the separation column was rinsed with 500 Buffer collected the effluent to get ALDH1A1− cells. ALDH1A1+ cells were removed from the separator and flushed with 1 mL Buffer to collect the outflow.

#### MTT Assay

After DU145, ALDH1A1+, ALDH1A1− cells adherence for 0, 24, 48, 72, 96 h and ALDH1A1+ cells treatment with drugs for 48 h, the culture medium was replaced with 0.5 mg/mL MTT solution in fresh medium and incubated for another 4 h. The supernatant was discarded while the formazan was resolved in 100 μL DMSO. Optical density (OD) values were read at a wavelength of 570 nm by Microplate reader replaces (ELX-800, BIOTEK, United States). All results were repeated for three times.

#### Quantification of ALDH1A1+ Cells by Flow Cytometry

The cells were washed twice with PBS and collected by centrifugation at 300 *g* for 5 min. Hundred μL PBS resuscitation cells containing ALDH1A1 antibody were added and incubated for 30 min in the dark. And cells were collected by centrifugation at 300 *g* for 5 min, the supernatant was discarded, washed with PBS for two times, centrifuged at 300 *g* for 5 min, the supernatant was discarded, 500 μL staining buffer was added to each tube of cell samples. Finally, cells were stored in ice bath to avoid light, and then enumerated by flow cytometry.

#### Migration Assay

Cells were seeded in 24-well plate at the density of 1 × 10^3^/well at 37°C and 5% CO_2_ in the incubator for 24 h. Wash the Transwell chamber twice with PBS. Fix 4% paraformaldehyde for 20 min at room temperature, dye 0.5% crystal violet solution for 5 min, rinse with distilled water. The number of cells migrating to the subcellular membrane was counted under an inverted microscope (200×). Five fields were selected for cell count for each well, and the mean number was taken as the cell number of migration of the well.

#### Invasion Assay

The Transwell chamber was placed into a 24-well plate, coated with 40 μL Matrigel prediluted onto the compartment membrane and the gel was placed in an incubator at 37°C for 2 h to solidify. The coated Transwell chamber was placed into a 24-well plate, and 800 μL of culture medium containing 30% FBS was added to the lower chamber. Two hundred μL cell suspensions were added to the upper chamber, and the cell Numbers were 1 × 10^4^/well at 37°C and 5% CO_2_ in the incubator for 24 h. Transwell chamber was washed twice with PBS and fixed 4% paraformaldehyde for 20 min at room temperature, dye 0.5% crystal violet solution for 5 min, rinse with distilled water. The number of cells invading to the subcellular membrane was counted under an inverted microscope (200×). Five fields were selected for cell count for each well, and the mean number was taken as the cell number of invasion of the well.

#### Plate Clonality Assays

Cells were plated in plates at the density of 100/plate and cultured at 37°C with 5% CO_2_ for 2 weeks. After colony formation, the culture medium was discarded, washed with PBS for two times, fixed with paraformaldehyde for 20 min, stained with Reggie’s complex dye for 5 min, and washed with PBS for excess Reggie’s complex dye.

#### Sphere Formation Assay

The cells were inoculated in 500/well in 6-well ultra-low adsorption cell culture plate with serum-free rpmi-1640 medium (adding 20 ng/mL EGF, 10 ng/mL bFGF, 5 nm glutamate, 1% cyn-streptomycin, 0.2% BSA, 1 × B27 without vitamin A, 1 × insulin iron-selenium transfer protein). The culture medium was changed every 3–4 days, and the formation of cell pellets was observed for 2–3 weeks.

#### Immunocytochemistry

Expression of ALDH1A1 was assessed by immunocytochemistry. Briefly, the cells were incubated with 0.1% TritonX-100 for 20 min. After incubated in 3% hydrogen peroxide for 15 min, sections were blocked with serum for 15 min at room temperature. And then incubated with rabbit anti-goat (1:100) antibodies overnight at 4°C. After that, cells were incubated with appropriate secondary antibodies, labeled with horseradish peroxidase and then stained with DAB & Hematoxylin.

### Animals and Treatment

Six-week-old female BALB/c nude mice were bought from Beijing HFK Bioscience Co. Ltd. [SCXK(Jing)2014-0004]. They were given free way to get food and water with humidity of 50 ± 10% at 23 ± 2°C or a 12 h light/dark cycle. The mice were randomly divided into groups: Control group and silybin treatment group. A logarithmic growth phase of ALDH1A1 + prostate cancer cells and Silybin-treated (200 μM) ALDH1A1+ prostate cancer cells were selected to prepare a cell suspension. 0.2 mL cells containing 1 × 10^6^ cells were taken and inoculated into the right armpit subcutaneously of mice. After waiting for the formation of the tumor in nude mice, record the tumor volume every 5 days. Mice were sacrificed after 5 weeks, and tumor tissues were collected for subsequent experiments.

### Real-Time PCR Analysis

The expression of ALDH1A1 mRNA was detected by Real-time PCR 24 h after transfection. Firstly, total RNA was extracted by Trizol method. Then, RNA concentrations in each sample were determined using an ultraviolet spectrophotometer (NANO 2000, Thermo, United States). The RNA samples obtained above were reverse-transcribed to obtain the 20 μL cDNA according to the manufacturer’s specifications. Fluorescence quantitative analysis was carried out with fluorescence quantifier (Exicycler 96, BIONEER, United States). The primers were shown as Table 2. The 2^–ΔΔCt^ method to calculate the relative expression of the purpose gene mRNA.

**TABLE 2:** PCR primer sequence.

Gene	Primer sequence
ALDH1A1 F	GGCAGCCATTTCTTCTCA
ALDH1A1 R	TGTCCAAGTCGGCATCAG
β-actin F	ACCCTGAAGTACCCCATCGA
β-actin R	CAAACATGATCTGGGTCATCT

### Western Blotting

Western Blot was used to detect to ALDH1A1, RARα, Ets1 and MMP9. The proteins were extracted with radio immunoprecipitation (RIPA) buffer and the concentration was quantitated by BCA assay. The total protein (100 μg) were separated on 10% resolving SDS – PAGE gel and 5% stacking gel, and transferred to PVDF membrane. Next, membranes were blocked with 5% skim milk in Tris – buffered saline containing 0.1% Tween – 20 (TBST) for 1 h, incubated with the primary antibody overnight at 4°C, and then incubated with horseradish peroxidase – conjugated secondary antibody after washing with TBST. Membranes were visualized using an enhanced chemiluminescence (ECL) detection system. The density of each band was estimated using the Image Lab software. All target proteins were normalized against the loading control β-actin.

### Statistical Analysis

Each experiment was performed at least three times, and all results were presented as means ± SD. Results were analyzed with IBM SPSS Statistics 19.0 (SPSS Inc., NY, United States). Significant differences were determined by a Student’s *t*-test and one-way analysis of variance (ANOVA) (^∗^*P* < 0.05 or ^∗∗^*P* < 0.01).

## Results

### Silybin Target Screening

**FIGURE 1:**
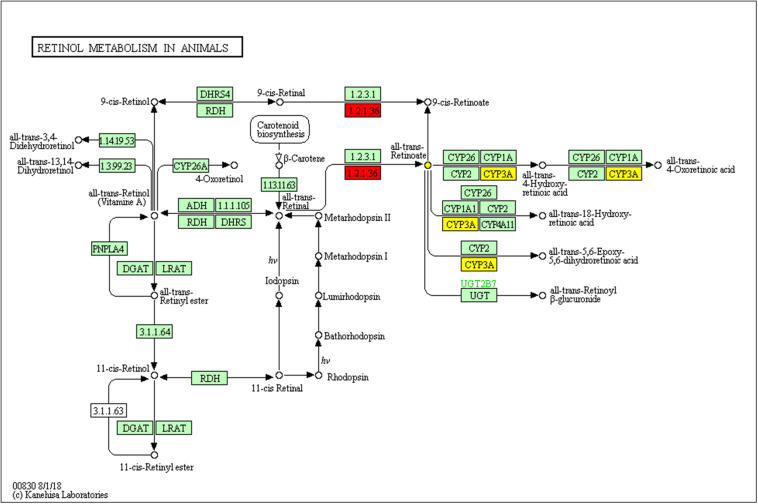
The local area (subpathway) of retinol metabolism pathway is highly correlated with ALDH1A1. The rectangles represent the enzymes and the circles represent compounds. The enzymes encoded by ALDH1A1 are marked with red. The red and yellow nodes represent the subpathway of the pathway.

Firstly, we used bioinformatics methods to analyze of the new target of Silybin. There were 26 active targets which are possible targets of Silybin, which obtained from PubChem database. Then the NCBI-Gene ID for each gene was entered into the Subpathway-GM system, and a sub-pathway of the retinol metabolism was identified. The result shown that the prostate cancer stem cell marker ALDH1A1 (EC 1.2.1.36) (Red nodes in Figure 1) are key enzyme in the sub-pathway.

### The Correlation Among ALDH1A1 and RARα and Ets1 Expression in Prostate Cancer Cells

**FIGURE 2:**
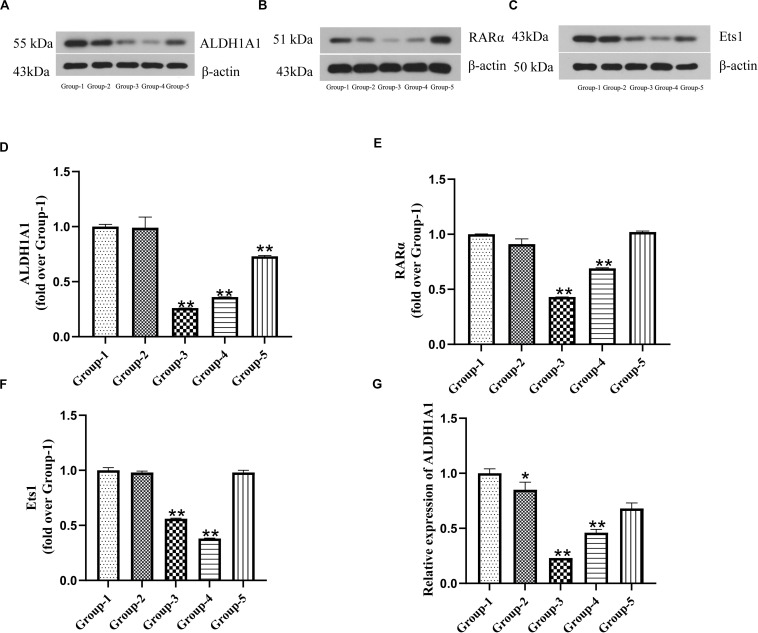
The expression of ALDH1A1 and RARα as well as Ets1 were positive correlation in prostate cancer cells. Group-1 was Untreated group, Group-2 was Negative matched control transfection group, and Group-3, -4, -5 were Transfection group (Group 3–5 were transfected with siRNA-1, siRNA-2, siRNA-3). The expressions of ALDH1A1 **(A)**, RARα **(B)**, Ets1 **(C)** were detected by Western blotting, Statistical analysis on them **(D–F)**. **(G)** Relative expression of ALDH1A1 mRNA was examined by RT-PCR. Data are expressed as the mean ± SD of three independent experiments. *P < 0.05 and **P < 0.01 compared with group-1.

To characterize the correlation of ALDH1A1, RARα and Est1, we measured the expressions of them after DU145 cells transfection by Western blotting and RT-PCR. The results showed that the expression of ALDH1A1 proteins and mRNA in transfection groups (siRNA-1, siRNA-2, siRNA-3) were significantly reduced compared with untreated group (*P* < 0.01) (Figures 2A,D,G). In the meanwhile, the productions of RARα and Est1 in transfections were also lower than untreated group which were transfected by siRNA-1 and siRNA-2 (*P* < 0.01), but it was no statistical difference by siRNA-3 (*P* > 0.05) (Figures 2B,C,E,F). Therefore, siRNA-1 was chosen to use in the subsequent experiments. The results indicated ALDH1A1 expression was positively correlated RARα with Ets1.

### ALDH1A1(+) (−) Cell Sorting and Biological Function

**FIGURE 3:**
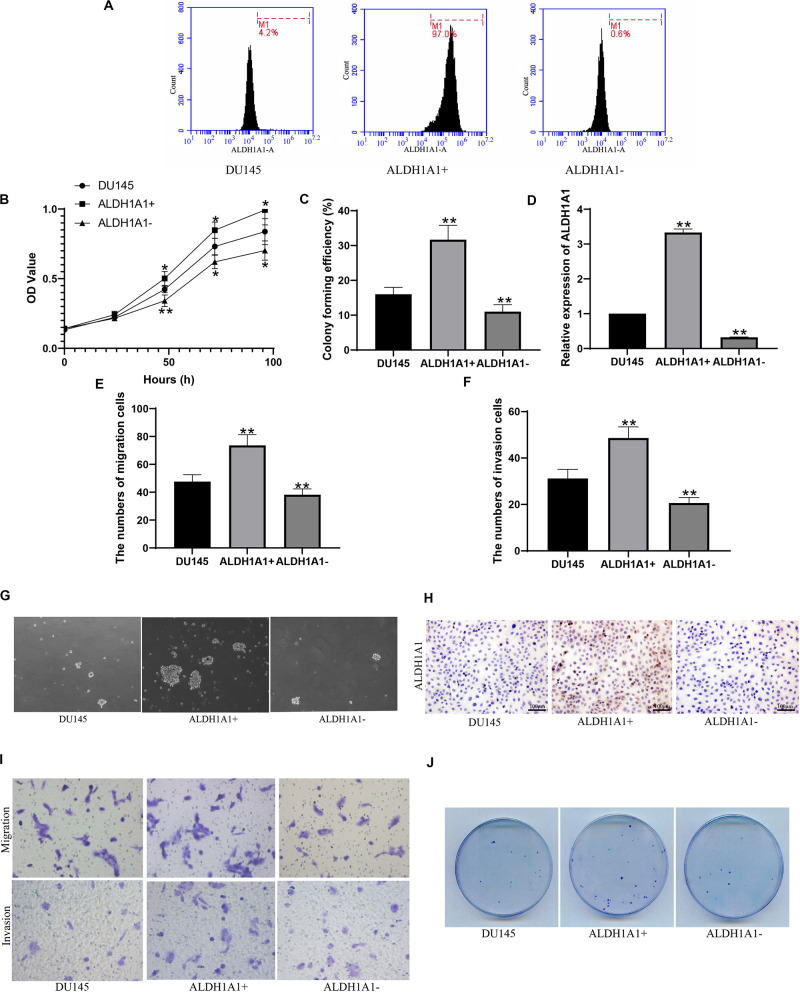
Screening and identification of ALDH1A1+ prostate cancer stem cell-like cells and their biological functions *in vitro*. Flow cytometry detected the expression of ALDH1A1 positive cells **(A)**. The cells proliferative capacity was detected at 0, 24, 48, 72, 96 h by MTT assay **(B)**. Plating efficiency was tested by plate clonality assay **(C)**, and was colony forming efficiency was calculated **(F)**. The expression of ALDH1A1 was detected by Immunocytochemistry (H, 200×, brown yellow granules indicate positive reaction). miR-ALDH1A1 was detected by Rt-PCR **(D)**. The images of Transwell assay were recorded **(I)** which revealed the numbers of migration **(E)** and invasion **(F)** cells. To detected assess the presence and self-renewal ability of cells by sphere-formation Assay **(G)**. The expression of ALDH1A1 were detected with immunohistochemistry **(H)**. Plate clonality was used to detect the colony ability of DU145 cells **(J)**. Data are expressed as the mean ± SD of three independent experiments. *P < 0.05 and **P < 0.01 compared with DU145.

Flow cytometry was used to sort and identify the ALDH1A1(+) cells. It was showed that cells containing ALDH1A1 gene were 4.2% in DU145 cells, 97.0% in ALDH1A1(+) cells and 0.8% in ALDH1A1(−) cells, respectively (Figure 3A). Then MTT assay results showed that ALDH1A1(+) cells group exhibited higher cell proliferative capacity than DU145 cells, but cell proliferative capacity of ALDH1A1(−) cells were obviously reduced compared to DU145 cells, after incubation 24–96 h (Figure 3B). And we also detected the proliferation ability by plate clonality which indicated that colony forming efficiency was higher in ALDH1A1(+) cells, but lower in ALDH1A1(−) cells, than in DU145 cells (Figures 3C,J). The results of RT-PCR verified that ALDH1A1 mRNA expression level in ALDH1A1(+) cells were significantly increased while was significantly reduced in ALDH1A1(−) cells compared to DU145 Cells (Figure 3D). After Transwell assay, the abilities of migration and invasion in ALDH1A1(+) cells were strengthened, whereas the abilities in ALDH1A1(−) cells were weakened, in comparison to DU145 cells (Figures 3E,F,I). Furthermore, we examined the protein of ALDH1A1 by immunocytochemistry and it was showed that the protein of ALDH1A1 was largely expression in ALDH1A1(+) cells but little expression in either ALDH1A1(−) cells or DU145 cells (Figure 3H). As is shown in Figure 3G, the number of ALDH1A1(+) cells continued to increase until the cells formed small spheres and many formed dense spheres, but no obvious globular formation was observed in ALDH1A1(−) cells after 2–3 weeks of cells inoculation.

### The Effect of Silybin on ALDH1A1(+) Cells

**FIGURE 4:**
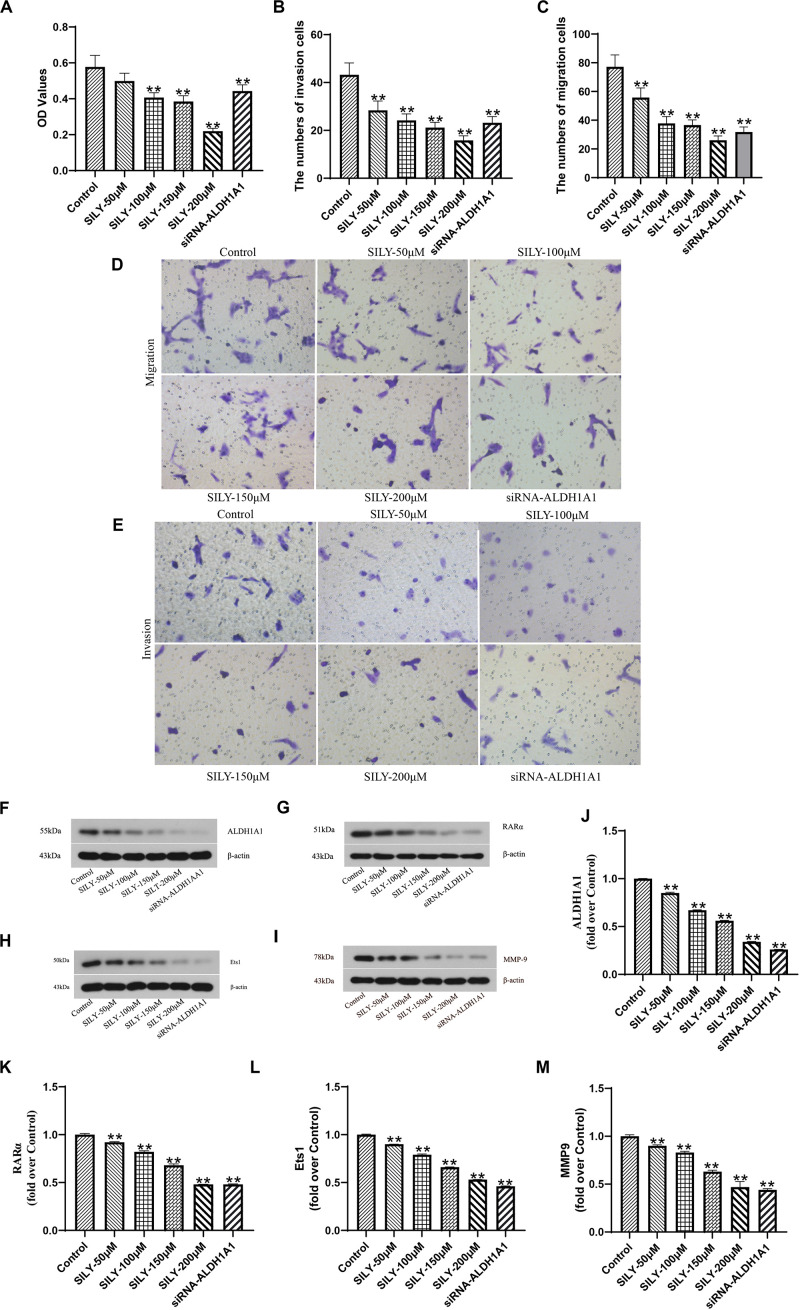
Biological function of Silybin on DU145 cells. The cells proliferative capacity was assessed by MTT assay **(A)**. The images of Transwell assay were recorded **(D,E)** which revealed the numbers of invasion **(B)** and migration **(C)** cells. The expressions of ALDH1A1 **(F)**, RARα **(G)**, Ets1 **(H)**, MMP9 **(I)** were detected by Western blotting, Statistical analysis on them **(J–M)**. Data are expressed as the mean ± SD of three independent experiments. *P < 0.05 and **P < 0.01 compared with Control group.

Having known that ALDH1A1(+) cells had high ability of proliferation, migration and invasion based on the above experiments. We further investigated whether Silybin inhibited prostate cancer stem cells growth *in vitro*. MTT assay showed that the cells’ ability to proliferate was inhibited after treated with different concentrations (100, 150, 200 μM) for 24 h (Figure 4A). And Silybin also inhibited cell migration and invasion according to the Transwell assay (Figures 4B–E). Then, the expressions of ALDH1A1, RARα, Ets1 were detected by Western blotting and the results showed that Silybin at the concentrations of 50–200 μM could reduce the productions of ALDH1A1, RARα and Ets1 (Figures 4F–M). Collectively, these results indicated that Silybin could inhibit prostate cancer by downregulating the expressions of ALDH1A1, RARα and Ets1 *in vitro*.

**FIGURE 5:**
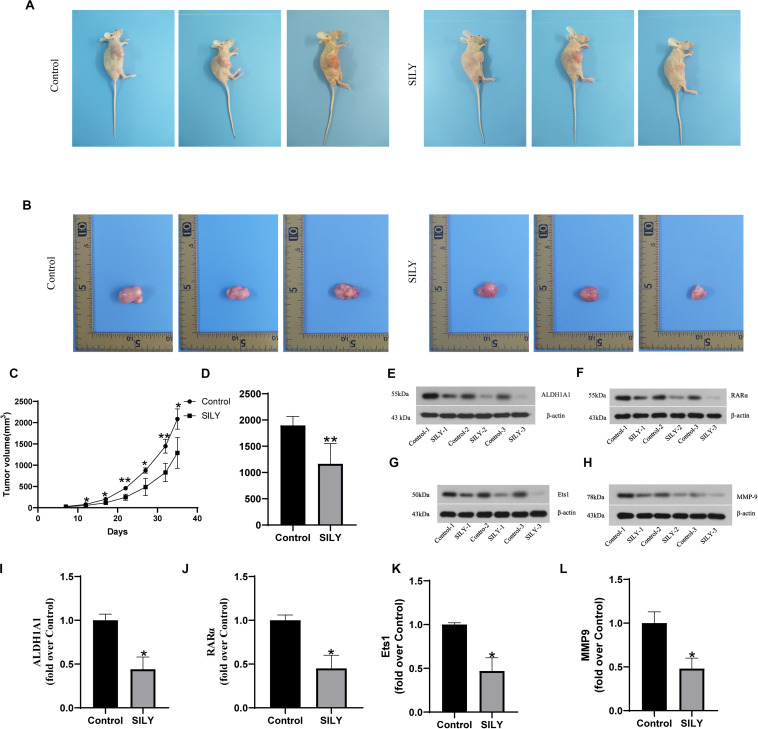
Biological function of Silybin *in vivo*. The picture of nude mice **(A)** and tumor **(B)** as well as the tumor weight **(D)** were recorded after sacrifice. And the tumor volume was detected after tumor formation in nude mice every 5 days **(C)**. The expressions of ALDH1A1 **(E)**, RARα **(F)**, Ets1 **(G)**, MMP9 **(H)** were detected by Western blotting, Statistical analysis on them **(I–L)**. Data are expressed as the mean ± SD of three independent experiments. *P < 0.05 and **P < 0.01 compared with Control group.

To further verify the antitumous role in prostate cancer, the mice models with implanted sarcoma were constructed (Figure 5A). And we recorded the tumor volume every 5 days after the tumor formed. The results showed that tumor volume in control group were greater than Silybin group after day 10 and the result was maintained until the mice sacrifice (Figures 5B,C). Also, tumor weight in control group was heavier than Silybin group (Figure 5D). According to the Western bolt results, the Silybin group exhibited lower expressions of ALDH1A1, Ets1, RARα and MMP9 than in control group (Figures 5E–L). Concluded from the above experiments, Silybin could reduce the levels of ALDH1A1, Ets1, RARα and MMP9 to inhibit the tumor growth *in vivo*.

## Discussion

In this study, we firstly screened out the ALDH1A1, which was highly correlated with subpathways of the Silybin risk metabolic pathway by bioinformatics method and then investigated the effect of ALDH1A1 expression on proliferation, migration and invasion of prostate stem cancer. And we further explored the mechanism of Silybin in inhibiting prostate cancer *in vitro* and *vivo*.

It wound greatly advance our understanding of tumor biology of prostate to develop the specific makers of prostate cancer. Recent studies have revealed that aldehyde dehydrogenase1A1 (ALDH1A1), an aldehyde oxidase that can degrade toxic aldehydes in cells to maintain a stable environment in the cell, was a marker for malignant prostate stem cells and predictor of prostate cancer patients’ outcome (Yoshida et al., 1992; Li et al., 2010). A clinical research indicated that the positive expression of ALDH1A1 in 163 cases of prostate cancer tissue was significantly increased in secretory carcinoma epithelioid cells and neuroendocrine tumor cells and the cells with positive expression had strong clonality and tumorigenicity (Li et al., 2010). In this study, we searched for possible targets of silybin from PubChem database and obtained 26 active targets. And the results of target-based screening by Subpathway-GM indicated that ALDH1A1 was the potential target for Silybin in the treatment of prostate cancer and it could regulate the synthesis of all trans-retinoic acid as well as 9-*cis*-Retinoate which could inhibit the cancer cell-stimulated proliferation and differentiation of the pro-tumoral macrophages and to act as a tumor suppressor gene in the prostate cancer (Jiang et al., 2006; Tsagozis et al., 2014). Hellsten et al. (2011) proved that ALDH1A1(+) prostate cancer cells showed cancer stem cell-like characteristics such as increased self-renewing and colony forming capacity and tumorigenicity. Therefore, we successfully isolated the ALDH1A1(+) cells from DU145 cells by using magnetic bead cell sorting (MACS) to investigate the biological function. And results showed that ALDH1A1 promoted the proliferation, migration and invasion ability of prostate stem cancer cells which indicated that ALDH1A1 was indeed a potential biomarker for prostate stem cancer cells.

Actually, ALDH1A1 as the major enzyme for retinoic acid synthesis, is responsible for converting aldehyde to acid (Landrier et al., 2017). Some research showed that ALDH1A1 could transform precursor retinaldehydes into retinoic acid receptor (RAR) which was a kind of steroid hormone receptor (Kiefer et al., 2012). Interestingly, deficiency of the ALDH1A1 gene also limited the formation of RAR (Shen et al., 2018). Besides, Hammond et al. (2001) demonstrated that antagonists of retinoic acid receptors (RARs) effectively inhibited the growth prostate carcinoma cells. In this study, we found that the expression of ALDH1A1 was obviously decreased along with the reduced levels of RARα after DU145 cells were interference by siRNA which was consistent with other studies. Previously, it had been exhibited that RARα could bind to E26 transformation-specific-1 (Ets1) promoter and induced the expression of Ets-1 mRNA and protein levels in cancer cells (Raouf et al., 2000; So and Crowe, 2000). And the overexpression of Ets1 were always associated with malignant biological features of prostate cancer (Li et al., 2012). Because Ets1 could affect extracellular matrix (ECM) degradation which was the premise of tumor cell metastasis (Cao et al., 2015). And Ets1 production also decreased after interference by siRNA. These results indicated that ALDH1A1 expression was positively correlated RARα with Ets1 in prostate cancer. ALDH1A1 promoted invasion and metastasis of prostate cancer by activating the RARα, which further activates Ets1.

Cancer stem cell hypothesis suggested that there are a few cells with stem cell-like characteristics in tumor tissues, which are able to proliferate, self-renew, differentiate in multiple ways, and promote the infinite proliferation of tumors and even the formation of new tumors (Mackillop et al., 1983). It has been proven that Silybin could inhibited the secretion of angiogenic factors and targeting the responses of endothelial angiogenic factors (motility, proliferation, survival, and differentiation) effectively for the treatment of prostate cancer cells (Singh et al., 2008). Our observation also exhibited that Silybin could suppress the proliferation, migration and invasion ability of prostate stem cancer cells. Besides, there is substantial evidence that decreased tumor cell proliferation and increased tumor cell apoptosis in mice significantly alleviated tumor growth (Chen et al., 2016). Regarding to tumor growth by measurements of tumor weight and volume, we found that tumor volume and weight of mice treated with Silybin was obviously reduced. As mentioned before, ALDH1A1 expression was positively correlated RARα with Ets1 and metalloproteinase 9 (MMP9) was one of the most important substance related to cancer invasion and metastasis (Nazir et al., 2019). It has been reported that inhibiting the expression of Ets1 gene could reduce proteins of the MMP9 secretion and significantly inhibit the invasion of PC3 cells (Kato et al., 2012). Yang et al. (2018) also indicated that the migration, invasion ability of PCa cells could be inhibited by downregulated the expression of MMP9. As expected, Silybin presented inhibition of ALDH1A1 expression and along with the lower expressions of RARα, Ets1 and MMP9. Therefore, these results consistently demonstrated Silybin had therapeutic effect on prostate cancer by downregulated the expression of ALDH1A1 *in vivo* and *vitro*.

In summary, firstly we screened out Silybin metabolic pathway was highly correlated with ALDH1A1. And then utilizing *in vitro* and *in vivo* experimental systems, we show that ALDH1A1 promoted invasion and metastasis of prostate cancer by activating the RARα, which further activated Ets1, MMP9 and Silybin inhibited the expression of ALDH1A1 in prostate cancer, thereby inhibiting the activation of RAR and preventing the activation of Ets1 to inhibit the growth and invasion of prostate cancer.

## Data Availability Statement

The raw data supporting the conclusions of this article will be made available by the authors, without undue reservation.

## Ethics Statement

The animal study was reviewed and approved by Institutional Animal Care and Use Committee (IACUC) of Heilongjiang University of Traditional Chinese Medicine.

## Author Contributions

YJ, JL, and DW conceived and designed the study. YJ, HS, and LJ performed the experiment. JL and DW guided the experimental operation. YJ, YQ, and DY drafted the manuscript. All the authors read and agreed to the manuscript.

## Conflict of Interest

The authors declare that the research was conducted in the absence of any commercial or financial relationships that could be construed as a potential conflict of interest.

## Footnotes

1 http://pubchem.ncbi.nlm.nih.gov/

2 https://github.com/chunquanlipathway/iSubpathwayMiner
