# Supplementary material for: Silybin Prevents Prostate Cancer by Inhibited the ALDH1A1 Expression in the Retinol Metabolism Pathway
Source: Front Cell Dev Biol. 2020 Aug 31;8:574394. doi: 10.3389/fcell.2020.574394 (PMC7487981; doi:10.3389/fcell.2020.574394)
Supplement: Supplementary file 1 [file Data_Sheet_1.PDF]

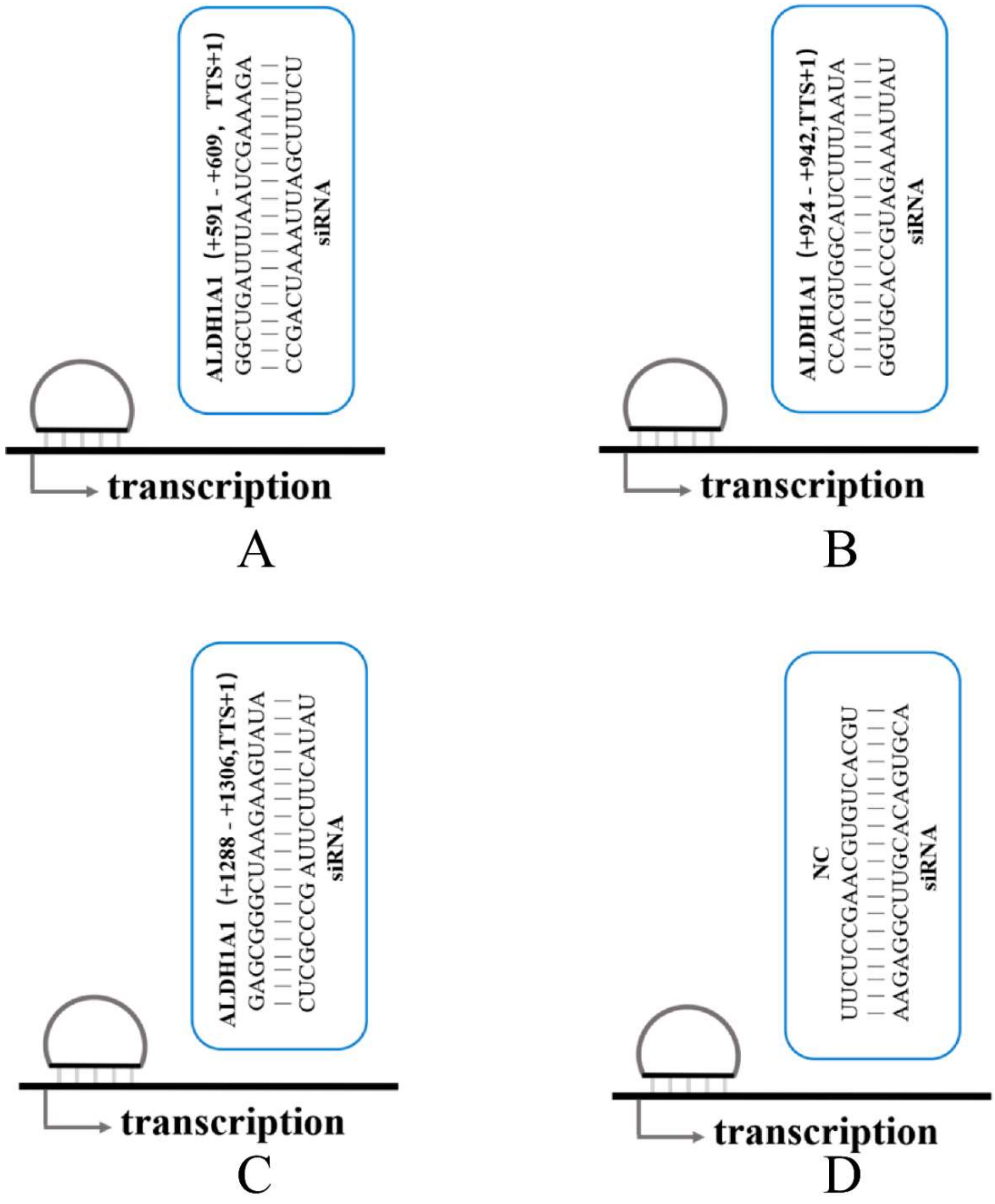

Supplementary Figure S1. RNA interference sequence diagram. (A) ALDH1A1-homo-591 siRNA. (B) ALDH1A1-homo-924 siRNA. (C) ALDH1A1-homo-1288 siRNA. (D) NC siRNA.
